# Description of the male of the Critically Endangered tarantula *Typhochlaena curumim* Bertani, 2012 (Araneae, Theraphosidae), with comments on tarantula trade and conservation

**DOI:** 10.3897/zookeys.938.51442

**Published:** 2020-06-04

**Authors:** Caroline Sayuri Fukushima, Pedro Cardoso, Rogério Bertani

**Affiliations:** 1 Finnish Museum of Natural History, University of Helsinki, P.O. Box 17 (Pohjoinen Rautatiekatu 13), 00014 Helsinki, Finland University of Helsinki Helsinki Finland; 2 Laboratório de Ecologia e Evolução, Instituto Butantan, Av. Vital Brazil, 1500 CEP 05503-900, São Paulo, Brazil Instituto Butantan São Paulo Brazil

**Keywords:** Aviculariinae, Brazilian Atlantic Rainforest, CITES, Mygalomorphae, pet trade, trafficking

## Abstract

The genus *Typhochlaena* C.L. Koch, 1850 consists of five small size arboreal tarantula species with remarkable colored abdominal patterns and a very restricted geographic range in Brazil. Here, we describe the male of *Typhochlaena curumim* Bertani, 2012, which was collected in an area of Brazilian Atlantic Rainforest. The new record extends the northern limit of the geographic range for both the genus and species. As *Typhochlaena* spp. are now especially popular and requested in the pet market, and because *T. curumim* is classified as Critically Endangered, we discuss the impacts of the international trade and other challenges on conservation of the genus.

## Introduction

The genus *Typhochlaena* C.L. Koch, 1850 was erected 170 years ago (Koch 1850) to include *Typhochlaena seladonia* (C.L. Koch, 1841) and *Typhochlaena caesia* (C.L. Koch, 1842) (now in *Caribena* Fukushima & Bertani, 2017). The first taxonomic revision and description of other species was only published in 2012 by Bertani (2012), who described *Typhochlaena amma* Bertani, 2012, *Typhochlaena costae* Bertani, 2012, *Typhochlaena paschoali* Bertani, 2012, and *Typhochlaena curumim* Bertani, 2012, all species endemic to Brazil. The genus is characterized by having a domed, short distal segment of the posterior lateral spinneret in all stages and a sternum, in adults, as long as wide and truncated behind (Bertani 2012). They are well known as small arboreal tarantulas with remarkable colored abdominal patterns, and they are becoming popular and increasingly requested in the pet trade, probably due to these characteristics.

*Typhochlaena curumim* is a species known from only three female specimens that were found under loose bark in an area of Atlantic Rainforest in the state of Paraíba, Brazil. Recently, males of the species were collected during arachnological expeditions in the state of Rio Grande do Norte, Brazil, and the aim of this paper is, therefore, to describe the previously unknown male of *T. curumim* and discuss problems of conservation as they relate to this genus.

## Material and methods

Specimens were deposited at the Museu Nacional, Rio de Janeiro (MNRJ) and at Museu de Zoologia da Universidade de São Paulo, São Paulo (MZUSP). A specimen from “Coleção de Arachnida” of the “Centro de Coleções Taxonômicas da Universidade Federal de Minas Gerais” (UFMG) was examined by photography.

The general format of the description is based on Bertani (2012). All the measurements are in millimeters (mm). A Leica M205C stereomicroscope with a DFC 450 camera attached, combined with a Leica LAS Montage and LAS 3D modules, was used to obtain images and measurements of small body parts. Large body parts, such as leg articles and carapace, were measured with digital Mitutoyo calipers with an error of 0.005 rounded up to two significant decimals. The measurements of legs and palps were taken on the dorsal aspect of the left side, unless appendages were lost, damaged, or obviously regenerated. Structures of the left side of the specimens were chosen for descriptions. Geographical coordinates and other locality details of the new records are redacted from this publication due to concerns that they may facilitate illegal collecting for the pet trade (Lindenmayer and Scheele 2017). Researchers interested in those records may obtain them directly from the institutions where the specimens are deposited. The map was made using the R package *red* – IUCN Redlisting Tools (Cardoso 2017). In the absence of better information, we assumed that the area within the convex hull polygon encompassing all known records had similar density of suitable habitat, i.e. Atlantic Rainforest remnants (according to SOS Mata Atlântica 2018), as the three states within it. As we expect the species to be present in several fragments within this polygon, we calculated the area of occupancy (AOO) as equal to extent of occurrence (EOO) multiplied by the proportion of Atlantic Rainforest in the three states. Terminology for urticating setae follows Cooke et al. (1972). Abbreviations: ALE = anterior lateral eye; AME = anterior median eye; ITC = inferior tarsal claw; PLE = posterior lateral eye; PLS = posterior lateral spinneret; PME = posterior median eye; PMS = posterior median spinneret.

## Taxonomy

### Typhochlaena curumim

Taxon classificationAnimaliaAraneaeTheraphosidae

Bertani, 2012

DA630454-A13A-5021-8938-AD691EECDCED

Figures 1 , 2−5 , 6−11 , 12

#### Diagnosis.

**Figure 1.**
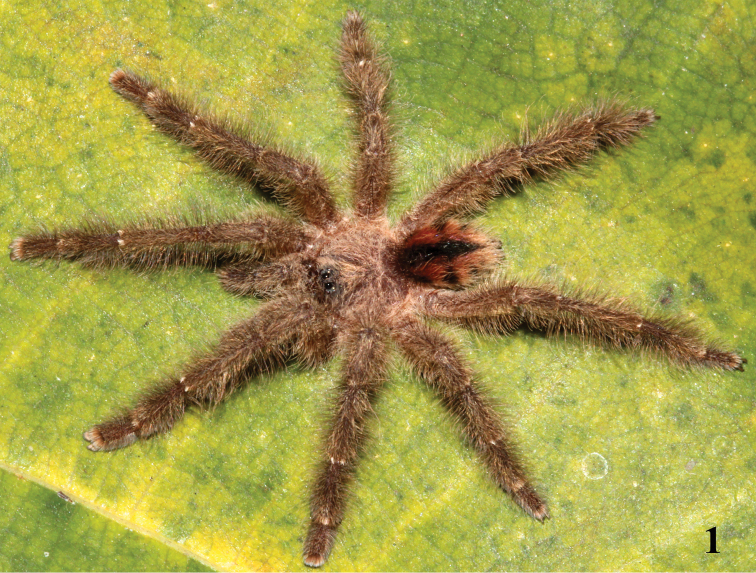
*Typhochlaena curumim* Bertani, 2012 habitus, male from southern Rio Grande do Norte, Brazil. Photo: Rogério Bertani.

Males of *T. curumim* resemble those of *T. seladonia* by having a long embolus, two or more times the tegulum length. Males of *T. curumim* differ from males of *T. seladonia* by the presence of shorter and broader embolus (≤ 2.5× length of tegulum in *T. curumim* versus > 3.5× in *T. seladonia*; 0.24 mm basal embolus width in *T. curumim* versus 0.1 mm in *T. seladonia*). They also differ by the brownish carapace and legs and abdomen dorsum with a black longitudinal stripe and lateral spots (Figs 1, 3). Apart from the long embolus (Figs 6–10), males of *T. curumim* differ from males of *Typhochlaena costae* and *Typhochlaena amma* by the characteristic abdominal color pattern (Figs 1, 3). Additionally, they differ from those of *T. costae* by the absence of long, curled, yellow setae over the carapace. For females, see the diagnosis by Bertani (2012).

#### Material examined.

Brazil, state of Rio Grande do Norte, locality data redacted: 1 male, S. N. Migliore leg., 19 April 2014, 19h58, over a bush leaf in a trail, ref. S90 (MNRJ 6915); 1 male, S. N. Migliore leg., 20 April 2014, 20h15, walking over a branch in a tree, ca 2 m high, ref. S102 (MZUSP 75781); locality data redacted: 2 males, W. Pessoa leg., 21–22 September 2014, walking over trees, < 2 m high, (MZUSP 75782); state of Ceará, locality data redacted: 1 male, L. S. Carvalho leg., 26–28 July 2013 (UFMG 15101), examined by photography.

#### Description.

**Male. MNRJ 6915.** Total length (without chelicera): 9.88. ***Carapace***: 5.18 long, 5.32 wide, 0.98× longer than wide; cephalic region not raised, thoracic striae inconspicuous. ***Chelicera***: 1.21 long. ***Abdomen***: 4.44 long, 3.74 wide. ***Spinnerets***: PMS, 0.70 long, 0.25 wide, 0.25 apart; PLS, 0.67 basal, 0.46 middle, 0.38 distal; midwidths 0.52, 0.47, 0.30, respectively.

***Fovea***: slightly procurved, shallow, 0.66 wide.

***Eyes***: eye tubercle 0.88 high, 0.81 long, 1.48 wide. Clypeus absent. Anterior row of eyes procurve. Posterior row of eyes slightly recurve. Eye sizes and interdistances: AME 0.35, ALE 0.35, PME 0.23, PLE 0.28, AME–AME 0.20, AME–ALE 0.19, AME–PME 0.05, ALE–ALE 0.20, ALE–PME 0.29, PME–PME 0.83, PME–PLE 0.05, PLE–PLE 1.11, ALE–PLE 0.2, AME–PLE 0.26.

***Maxilla***: 2.29 longer than wide. Cuspules: 28 spread over ventral inner heel.

***Labium***: 0.60 long, 1.04 wide, with 50 cuspules spaced by one diameter of each other on the anterior half. Labio-sternal groove shallow and flattened, with two slightly separate, large sigilla.

***Chelicera***: rastellum absent, basal segment with seven teeth and some small teeth on promargin.

***Sternum***: 2.45 long, 2.40 wide. Sigilla: three pairs, posterior and median rounded, less than one diameter from margin; anterior not visible.

**Figures 2–5.**
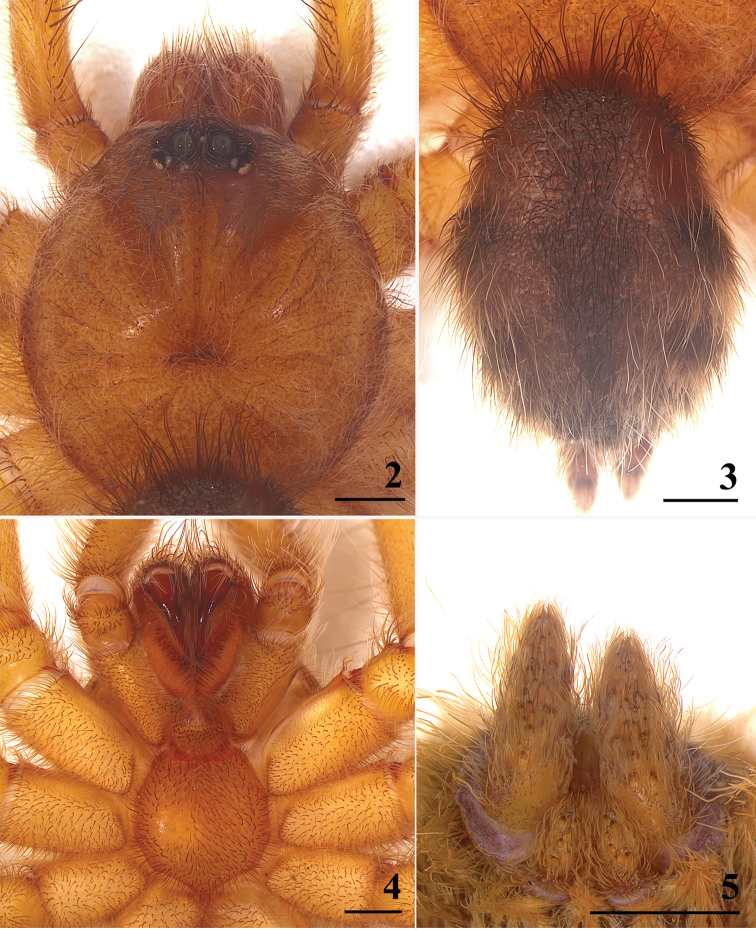
*Typhochlaena curumim* male (MNRJ 6915) **2** carapace, dorsal **3** abdomen, dorsal **4** maxillae, labium, sternum, and coxae **5** spinnerets, ventral. Scale bars: 1 mm.

***Legs*** (femur, patella, tibia, metatarsus, tarsus, total): I: 6.79, 3.22, 5.48, 4.43, 2.55, 22.47. II: 6.31, 3.02, 4.74, 4.54, 2.32, 20.93. III: 5.26, 2.55, 4.23, 3.80, 2.22, 18.06. IV: 6.62, 2.64, 4.80, 5.14, 1.97, 21.22. Palp: 3.24, 1.80, 2.97, ―, 1.09, 9.10. Midwidths: femora I–IV = 1.27, 1.18, 1.16, 1.02, palp = 0.82; patellae I–IV = 1.20, 0.98, 1.00, 1.05, palp = 0.84; tibiae I–IV = 0.75, 0.90, 0.84, 0.72, palp = 0.83; metatarsi I–IV = 0.63, 0.71, 0.77, 0.54; tarsi I–IV = 0.91, 0.73, 0.73, 0.54, palp = 1.05. Formula: I IV II III. Length leg IV to leg I: 0.94. Clavate trichobothria: two rows on distal 1/2 of tarsi I–IV. Scopula: Tarsi I–IV fully scopulate, IV divided by a wide band of setae. Metatarsi I–II on distal 2/3; III on distal 1/3; IV on distal 1/4. IV divided by setae. Scopula hairs longest at lateral areas of tarsi and metatarsi, giving spatulate aspect to articles. Spines absent on all legs and palps.

***Urticating setae***: type II (0.56–0.59 long) on the abdomen dorsum (Fig. 11).

**Figures 6–11.**
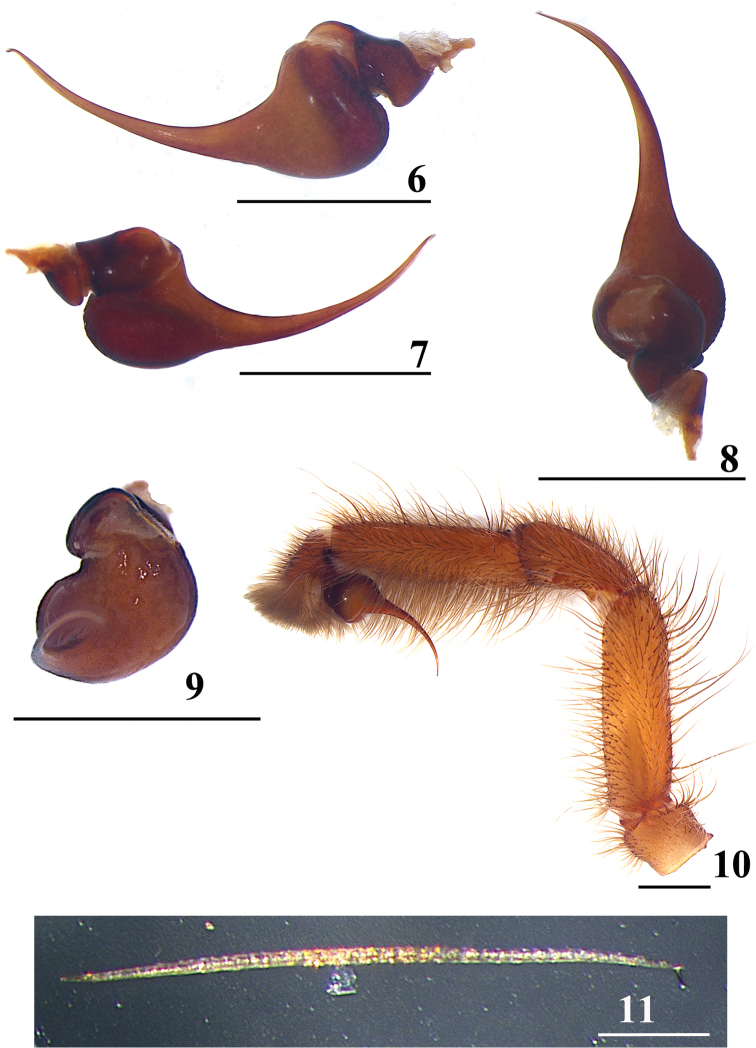
*Typhochlaena curumim* male (MNRJ 6915) **6−10** palpal bulb, left side **6** retrolateral **7** prolateral **8** dorsal **9** frontal **10** left palp, prolateral **11** urticating setae type II. Scale bars: 1 mm (**6–10**); 0.1 mm (**11**)

***Palp*** (Figs 6–10): globous bulb with small subtegulum and slightly developed prominence on tegulum. Embolus: not flattened, lacking keels, 1.62 long in retrolateral view (Fig. 6), about 2.5× length of tegulum. Proximal part not curved in frontal view (Fig. 9); thin distal width, tapering distally; basal, middle and distal width 0.24, 0.08, 0.01, respectively. Tegulum: 0.71 long, 0.4 high in retrolateral view (Fig. 6). Cymbium with two subequal lobes, lacking process on retrolateral lobe.

***Tibial apophysis***: absent. Metatarsus I straight.

***Color pattern*** (Figs 1–5): carapace and chelicerae dark brown with pale yellow long hairs on the margin of the carapace. Legs and palps dark brown, except for brown femora. Cephalic region, legs, palps, and chelicerae covered with long and abundant chestnut-brown setae. Coxae brown. Labium, sternum, and maxilla dark brown. Longitudinal stripes on femora, patellae, tibiae, and metatarsi inconspicuous. Distal femora, patellae, tibiae, and metatarsi rings whitish. Abdomen metallic reddish orange, dorsally with central longitudinal black stripe and three dark spots on each lateral.

#### Distribution.

**Figure 12.**
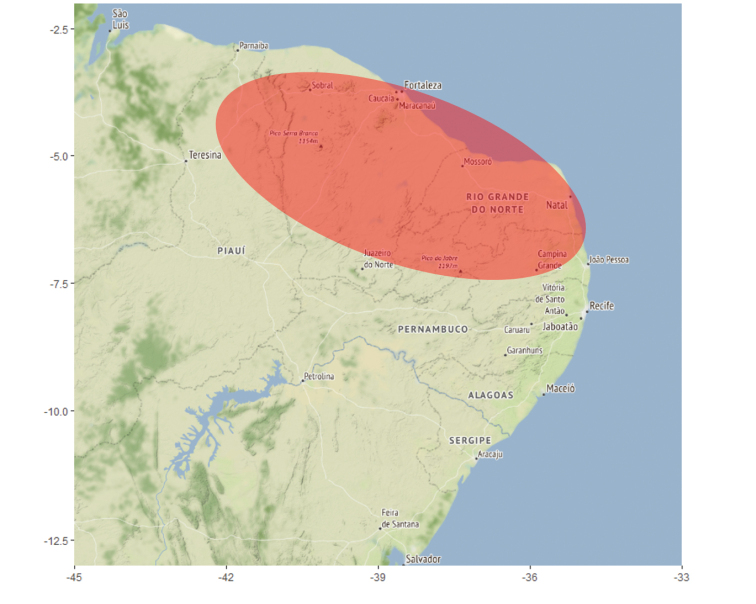
Approximate distribution of *Typhochlaena curumim* Bertani, 2012. A large spatial error has been introduced to avoid revealing exact localities.

In Brazil in the states of Rio Grande do Norte and Ceará (new records), and Paraíba (Bertani 2012), in remnants of Brazilian Atlantic Rainforest (Fig. 12).

#### Color pattern ontogeny.

There are no drastic ontogenetic changes of color pattern. Males have reduced lateral black stripes when compared to females and immatures.

#### Natural History.

Males from a more northerly site in Rio Grande do Norte (approximately at 6.02°S, 35.2°W) were resting on a leaf in a bush near a trail during the night, or high in a tree, walking on branches during the day. Males from a southern site in Rio Grande do Norte (approximately at 6.46°S, 35.0°W) were also found to be active at night in trees; one individual was seen at breast height and another at less than 2 m above the ground.

## Discussion

As in other genera of Aviculariinae, *Typhochlaena* species have a very restricted geographic range, with no overlap in the distribution among species (Bertani 2012). As proposed for species occurring in the Brazilian Atlantic Rainforest (Pellegrino et al. 2005), the geographic ranges of *Typhochlaena* species are mostly concordant with with river basins (Bertani 2012). The southernmost record for this genus is southern Espírito Santo; however, a new record of *T. curumim*, from northern Ceará, is the most northern for *Typhochlaena*.

Although two other aviculariine species, *Iridopelma hirsutum* Pocock, 1901 and *Pachistopelma rufonigrum* Pocock, 1901, have similar distributions to *T. curumim*, *T. curumim* is probably more affected by habitat loss, given its rarity (Bertani 2012). Moreover, the habits and ecological traits of *T. curumim* are poorly known, making it difficult to monitor and propose measures for its conservation. As *Typhochlaena* is the sister group of a large clade within Aviculariinae, conservation actions towards this species are equally applicable for preservation of the evolutionary diversity of the entire subfamily (Vane-Wright et al. 1991; Faith 1992).

Considering the whole genus as of 2012, only 40 specimens are deposited in zoological collections (Bertani 2012), and the current situation is most likely not much different. In contrast, the number of specimens detected in the pet trade is much higher. A brief online search revealed specimens of *Typhochlaena* spp. being sold in Germany, Spain, Czech Republic, Austria, South Africa, Poland, United Kingdom, Canada, and USA. Although Brazilian environmental law precludes the collection, possession, transport, export, and commercialization of Brazilian wildlife apart from exceptional cases (IBAMA 1998 – Lei de Crimes Ambientais 9605/98), theraphosid specimens are constantly smuggled out of the country to supply the pet trade.

The international pet trade is an important driver of biodiversity loss (Bush et al. 2014). However, since invertebrates are usually overlooked when formulating conservation actions and policies (Cardoso et al. 2011), tarantula trafficking has low priority for enforcement authorities. Loopholes in Brazilian environmental law, permeability of borders, and the ease of smuggling spiders internationally by “brown-boxing” specimens (i.e. sent in unlabeled packages by post) also contribute to the flow of tarantulas from Brazil to other points of the world. Once out of the country, many Brazilian specimens of tarantulas are sold in the pet trade. Although the presence of Brazilian tarantulas on the North American and European markets is not recent, records of seizures are very scarce (Caldas et al. 2018). Curbing tarantula trafficking is further complicated by the differing legislation and attitudes among the various countries. In the European Union, for example, it is allowed to acquire most non-CITES wildlife without restrictions, but in the USA the situation is quite different. The release of an official communication from the United States Fish and Wildlife Services (USFWS) applying the Lacey Act (http://www.thetkc.org/usfws-statement-regarding-contraband/) to a shipment of *Typhochlaena seladonia* of a well-known breeder demonstrates this difference. The Lacey Act requires US citizens to comply with laws enacted in foreign countries related to flora and fauna endemic to those countries (Lacey Act 18 USC 42-43, 16 USC 3371-3378), and all US citizens possessing or trading in imported Brazilian tarantula specimens, or their offspring, are violating US law because such activities are illegal in Brazil. Even though there was not any repatriation or re-introduction in the wild, the application of the Lacey Act to tarantula commercial shipments may be crucial in bringing attention to the pet trade, its association with wildlife trafficking, and in initiating an important discussion about the role of hobbyists, breeders, and traders in promoting or curbing this illegal activity.

*Typhochlaena curumim*, along with *T. seladonia*, was included in the Brazilian Red List of Threatened Species (ICMBio 2018). *Typhochlaena curumim* was classified as Critically Endangered, whereas *T. seladonia* was considered Endangered. The fact that *T. curumim* was known only from a single locality, an enclave of forest surrounded by dry areas, was a relevant reason for the assessors to consider the species as Critically Endangered. Although there are no data on the number of locations or if this species is severely fragmented, the inferred continuing decline in the extent and quality of habitat is still present and the new records presented here extend the species’ area of occupancy to 204 km2. This might contribute to a future change in classification from Critically Endangered to Endangered.

There are several difficulties in assessing the risk of extinction of invertebrates, mainly due to the scarcity of data on distribution and population size (Cardoso et al. 2011). Furthermore, invertebrates are normally described using one or very few specimens from a single locality; therefore, new species are frequently fit into the higher categories of threat after their first description. An example is *Phoneutria bahiensis* Simó & Brescovit, 2001, described in 2001 (Simó and Brescovit 2001) from four localities approximately 400 km apart and included in the Brazilian Red List in 2003 (MMA 2003). Just a few years later, the distribution of this species was considerably extended (Martins and Bertani 2007; Dias et al. 2011) and *P. bahiensis* was subsequently removed from the Red List (ICMBio 2014). Thus, it is necessary to pay attention to this peculiarity when assessing species for the Red List, as new data can rapidly discredit assessments of little-known species, as well as the whole list.

Despite the Brazilian Red List having no status of law, a species’ presence on the list increases the penalties for environmental crimes in Brazil by at least tenfold (MMA 2008). In this sense, it should discourage one to collect and to smuggle Red-Listed species. However, the penalties have not been sufficient to prevent international trafficking. The presence of a species in the trade can potentially affect wild populations, particularly in range-restricted species that have attracted international demand (Jansen and Leupen 2019), which is the case of *Typhochlaena* spp. A helpful conservation action would be the inclusion of *Typhochlaena* spp. in the global IUCN Red List, as this would draw the attention of international authorities and decision makers worldwide. Another important tool to regulate and to protect species from trade overexploitation is CITES, the Convention on International Trade in Endangered Species of Wild Fauna and Flora. The addition of all *Typhochlaena* spp. in CITES appendix I, which lists species threatened with extinction due to trafficking from the wild (and consequently prohibited from international trade unless the import purpose is non-commercial) (CITES 2020), would control or at least make tracking the rampant trade of the genus mandatory. This would be particularly relevant to the EU, as its lack of a consistent approach to legislation related to the trade in wild exotic pets (Endcap 2012) make it the primary destination of smuggled wildlife.

## Supplementary Material

XML Treatment for Typhochlaena curumim
